# Did COVID-19 impact perinatal outcomes differently in public and private maternity hospitals in Brazil?

**DOI:** 10.61622/rbgo/2025rbgo80

**Published:** 2025-10-21

**Authors:** Ellen Machado Arlindo, Renato Teixeira Souza, Maria Laura Costa, Jose Guilherme Cecatti, Edson Vieira da Cunha, Janete Vettorazzi

**Affiliations:** 1 Universidade Federal do Rio Grande do Sul Porto Alegre RS Brazil Universidade Federal do Rio Grande do Sul, Porto Alegre, RS, Brazil.; 2 Hospital Moinhos de Vento Department of Obstetrics and Gynecology Porto Alegre RS Brazil Department of Obstetrics and Gynecology, Hospital Moinhos de Vento, Porto Alegre, RS, Brazil.; 3 Universidade Estadual de Campinas Department of Obstetrics and Gynecology Campinas SP Brazil Department of Obstetrics and Gynecology, Universidade Estadual de Campinas, Campinas, SP, Brazil.; 4 Hospital de Clínicas de Porto Alegre Department of Obstetrics and Gynecology Porto Alegre RS Brazil Department of Obstetrics and Gynecology, Hospital de Clínicas de Porto Alegre, Porto Alegre, RS, Brazil.

**Keywords:** COVID-19, SARS-CoV-2, Pandemic, Pregnancy complications, Maternal health, Public health system, Perinatal care

## Abstract

**Objective:**

To compare maternal and perinatal outcomes in pregnant and postpartum women with severe acute respiratory syndrome coronavirus 2 (SARS- CoV-2) infection among admissions in public and private maternity hospitals before COVID-19 vaccination.

**Methods:**

We performed a secondary analysis of the REBRACO (in Portuguese, the Brazilian Network of COVID-19 During Pregnancy) initiative, a national multicenter cohort study in Brazil, considering pregnant and postpartum women with suspected or confirmed SARS-CoV-2 infections (from February 2020 to February 2021) in 15 maternity centers (2 private and 13 public facilities). Sociodemographic and obstetric characteristics were compared according to the type of hospital care. The clinical and laboratory findings and maternal and perinatal outcomes were compared between the two groups. The prevalence ratio and its 95% confidence interval for each predictor and outcome were calculated.

**Results:**

Of the 559 symptomatic cases tested, 289 confirmed COVID-19 cases were included, with 213 (72.7%) and 76 (27.3%) women in public and private hospitals, respectively. The frequency of SARS-CoV-2 infection did not differ significantly between the groups. Women treated at public hospitals had lower education levels (p<0.001), and 50% declared that their pregnancy was unplanned. We recorded 13 maternal deaths among women treated at public hospitals and no maternal deaths among pregnant women treated at private hospitals (p=0.024). Pregnant women in public hospitals had higher rates of fever (p=0.041), tachypnea (p=0.003), abnormal laboratory findings of liver enzymes (p=0.005), and severe acute respiratory syndrome (SARS) (p=0.014), and their neonates presented with more neonatal respiratory distress (p=0.020).

**Conclusion:**

Adverse maternal and perinatal outcomes were worse in the public hospital group, with increased rates of SARS and neonatal respiratory distress. The alarming difference in the number of deaths between patients treated in the public and private sectors highlights the urgency of better understanding the social determinants of health and calls the attention of leaders and policymakers to take action in mitigating their impact.

## Introduction

Severe acute respiratory syndrome coronavirus 2 (SARS-CoV-2) infection can be asymptomatic in approximately 75% of pregnant women. When symptomatic, 85% of pregnant women experience mild disease. Approximately 15% of cases progress to severe forms, with 4% and 3% of infected pregnant women requiring admission to the intensive care unit (ICU) and invasive ventilation, respectively.^(1)^ Furthermore, a study conducted in Washington consistently revealed a greater risk of severe COVID-19 infection and mortality among pregnant women compared to non-pregnant adults.^(2)^

Adaptations of the maternal immune system to allow for fetal growth result in an altered immune response to viral infections during pregnancy. The immunological response, respiratory response, coagulation response, and endothelial cell function act synergistically to determine the impact of infection on pregnancy.^(3)^ Maladaptive immune events featuring a delayed type 1 interferon response, increased neutrophil extracellular traps, excessive release of cytokines, lymphopenia, hyperinflammation, a hypercoagulable state, and vascular injury lead to COVID-19-associated complications. These processes may cause injury to the maternal–fetal interface, resulting in placental dysfunction. COVID-19 during pregnancy has been reported to be associated with a greater risk of poor prognosis and adverse perinatal events.^(4)^

Some characteristics, such as advanced maternal age, lower respiratory tract infection, radiological findings, lymphopenia, and increased inflammatory parameters at the time of hospital admission, are related to higher rates of adverse outcomes.^(4,5)^

Since the beginning of the pandemic in Brazil, the lethality of coronavirus disease 2019 among pregnant and postpartum women has been greater than that reported in developed countries.^(6)^ Understanding the clinical features of disease in this population and the impact of structural and social changes due to the pandemic on perinatal outcomes has been identified as a research priority.^(7)^

The impact of COVID-19 markedly varies in different countries worldwide, and the reasons for this diversity are multifactorial, including socioeconomic and geographic influences.^(8)^ The lack of public health models resembling the Brazilian health model has led to difficulties comparing the quality and capacity of adequate care for SARS-CoV-2 infections in pregnant women throughout the different pandemic scenarios.^(6)^

Knowledge of the different realities of Brazil can elucidate this disease and reduce maternal and perinatal morbidity and mortality. This study aimed to compare the characteristics and outcomes of pregnant and postpartum women admitted due to SARS-CoV-2 infection in the public and private sectors in different maternity centers in Brazil.

## Methods

This study was a secondary analysis of the Brazilian REBRACO Network, a national multicenter study that aimed to elucidate the impact of COVID-19 on pregnancy in a Brazilian obstetric population. Pregnant women with suspected and/or confirmed SARS-CoV-2 infection were followed up, and maternal and perinatal data were collected in public and private hospitals comprising 15 maternity centers across the country. Data for outpatient and hospitalized women were collected from February 1, 2020, to February 28, 2021, before the implementation of COVID-19 vaccination.

The inclusion criteria were pregnant women who attended the obstetrical services of the participating centers and presented with flu-like symptoms and confirmed SARS-CoV-2 infection.

Laboratory testing and/or radiological pulmonary findings confirmed the COVID-19 diagnosis. Participants were tested for SARS-CoV-2 infection according to the local availability of testing and subjected to laboratory exams and/or computed tomography (CT) scans following local clinical protocols.

Women who had a negative real-time polymerase chain reaction or rapid test, those who were not tested, and those who did not have ground-glass opacity-compatible abnormalities on CT scans submitted to radiological investigation were considered negative for SARS-CoV-2 infection and were excluded from the study.

To evaluate pregnancy outcomes, only women who tested positive for COVID-19 and whose follow-up was considered successful (available childbirth information and COVID-19 status) were included in the analysis. Women with unavailable late pregnancy outcomes (unknown mode of delivery and gestational age at delivery), postpartum women at enrollment, and women with ongoing pregnancy at the time of this analysis were excluded.

We collected data on sociodemographic factors, pregnancy, medical history characteristics, and the initial clinical presentation of COVID-19. After the clinical presentation of COVID-19, the women were followed up until childbirth and the postpartum period.

The women from 15 maternity hospitals in four Brazilian regions were divided into public and private hospital groups. The public hospital group included the following hospitals: Woman's Hospital of the University of Campinas, São Paulo (SP); Hospital of Jundiai School of Medicine (SP); Clinical Hospital of Porto Alegre, Rio Grande do Sul (RS); Minas Gerais (MG); Maternity Hospital of Federal University of Ceara (CE); Hospital of Federal University of São Paulo (SP); Jorge Rossmann Regional Hospital of Itanhaem (SP); Hospital of Federal University of São Carlos (SP); Sumare State Hospital (SP); Hospital of Federal University of Minas Gerais (MG); Fernandes Figueira Institute, Rio de Janeiro (RJ); Hospital of the Sao Paulo State University in Botucatu (SP); Hospital of Federal University of Pernambuco (PE); and Santa Casa de Misericórdia of Para (PA). The private hospital group included UNIMED Maternity of Belo Horizonte (MG) and Moinhos de Vento Hospital of Porto Alegre (RS).

As no prior data were available in the literature, the statistical power was calculated a posteriori for the composite outcome (maternal death, ICU admission, and SARS). Considering a significance level of 5%, and the occurrence of this outcome in 25.4% of public hospitals and 10.5% of private hospitals, a statistical power of 83.37% was obtained.

The study data were collected and managed via REDCap®15 (Research Electronic Data Capture) tools hosted at the CAISM/Unicamp server, the coordinating center. The research collaborators had hierarchical and clustered access to the system. The data were properly anonymized and kept confidential.

We reported the number of women with positive COVID-19 results, the proportion of cases investigated (COVID-19 tests performed), and the number of cases confirmed for all participants during the study period.

We compared sociodemographic data (age, education, marital status, pregestational body mass index [BMI], and region), pregnancy conditions (multiple pregnancies, parity, planned or unplanned pregnancy, and type of prenatal insurance), and medical condition characteristics (alcohol use, asthma, chronic kidney disease, diabetes, hypertension, and smoking) between women who attended public and private hospitals for childbirth. To assess obstetric outcomes, the following variables were evaluated: mode of birth, miscarriage, fetal death, prematurity (childbirth <37 weeks) preeclampsia (new onset of hypertension, blood pressure ≥ 140/90 mmHg in two or more measures after 20 weeks of gestation with proteinuria or other laboratory or clinical signs of organ dysfunction), birth weight (adequacy of birth weight according to gestational age using the GROW customized chart), Apgar score (<7 at 5 min), respiratory distress, admission to the neonatal intensive care unit (NICU), and neonatal death.

Information regarding the severity of COVID-19 included SARS, admission to the NICU, need for intubation and prone positioning, renal impairment, maternal death, and severe maternal outcomes (SMOs). SMOs were defined as any of the following: SARS, admission to the ICU, or maternal death.

The data were subsequently dichotomised into public and private groups. Qualitative variables were compared using chi-square or Fisher's exact tests depending on the number of participants to assess the statistical significance of differences between groups. Pearson's and Spearman's correlations were used to analyze quantitative variables based on normal distribution. To determine the associations between COVID-19 and pregnancy outcomes in public and private hospitals, we estimated unadjusted risk ratios with 95% confidence intervals.

All the statistical analyses were conducted via IBM SPSS Statistics for Windows, Version 26.0 (IBM Corp., Armonk, NY).

The study protocol adhered to the Declaration of Helsinki amended in 1964 and was approved by the institutional review board of the coordinating center and by each participating center (Certificado de Apresentação de Apreciação Ética – CAAE 31591720.5.0000.5404, approval number:4.047.168). The Strengthening the Reporting of Observational Studies in Epidemiology guidelines were followed for the implementation and reporting of the study. All the included women received detailed information regarding the study and provided their informed inference for study participation.

## Results

**Figure 1:**
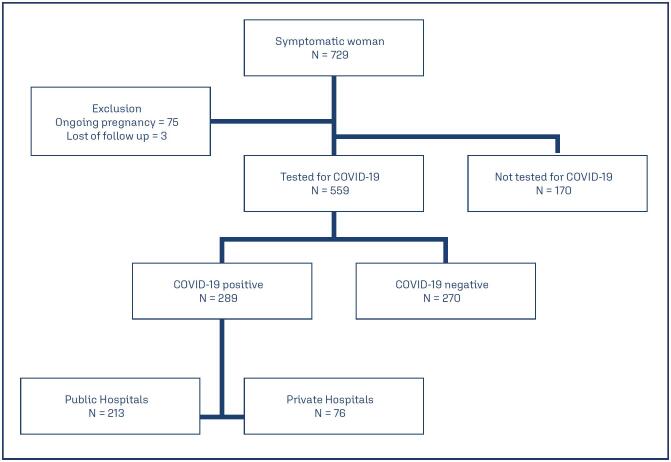
Flowchart of the inclusion of women in this study

From the initial 729 symptomatic pregnant women in the study database, this analysis considered all confirmed positive cases with data on pregnancy outcomes, including 289 cases, with 213 women from public hospitals and 76 women from private hospitals. Figure 1 presents a flowchart of the included cases, detailing the exclusion criteria. The sociodemographic and obstetric data are summarized in table 1. The women cared for in the public healthcare system had lower education levels (none/primary incomplete) (11.4% vs. 0%) and a higher rate of unplanned pregnancies (50% vs. 28.9%) compared to those in private hospitals. In the private hospital group, first pregnancy represented a greater proportion of public insurance used for prenatal care (80.3% vs. 72.2%), and the women had a lower BMI (40.9% vs. 25.7%) and more often had a partner (61.8% vs. 76.3%) than those in did the public hospital group. Furthermore, 31.8% of the women in the public hospital group and 46.1% in the private hospital group were primigravidae.

**Table 1:** Sociodemographic data, previous clinical data, and characteristics of the symptomatic women with confirmed COVID-19 according to the type of hospital care

Women's characteristics		Public hospitals n(%)	Private hospitals n(%)	p-value
Maternal age				0.105
	≤19	14(6.6)	1(1.3)	
	20–35	154(72.3)	53(69.7)	
	>35	45(21.1)	22(28.9)	
Skin color^*^				0.483
	White	114(54.5)	45(59.2)	
	Non white	95(45.5)	31(40.8)	
Schooling**				<0.001
	None/primary incomplete	20(11.4)	0(0.0)	
	Primary/secondary	118(67.0)	20(31.7)	
	College or higher	38(21.6)	43(68.3)	
	First pregnancy***	67(31.8)	35(46.1)	0.026
	Multiple pregnancy	9(4.2)	1(1.3)	0.464
	With partner****	128(61.8)	58(76.3)	0.023
Health insurance of antenatal care*****				<0.001
	Public	186(94.9)	0(0.0)	
	Private	10(5.1)	74(100)	
Pregnancy status at enrolment				0.368
	Pregnant	195(91.5)	72(94.7)	
	Postpartum	18(8.5)	4(5.3)	
Trimester of COVID-19 diagnosis				0.079
	1st trimester	23(10.8)	13(17.1)	
	2nd trimester	48(22.5)	25(32.9)	
	3rd trimester	124(58.2)	34(44.7)	
	Postpartum	18(8.5)	4(5.3)	
	Chronic hypertension	18(8.5)	7(9.2)	0.840
	Obesity	54(40.9)	18(25.7)	0.046
	Gestational diabetes	5(2.3)	1(1.3)	0.588
	Asthma	18(8.5)	3(3.9)	0.194
Region				<0.001
	North/Northeast	45(21.1)	0(0.0)	
	Southeast	143(67.1)	42(55.3)	
	South	25(11.7)	34(44.7)	
	Unplanned pregnancy******	87(50.0)	22(28.9)	0.002

Missing information for:

* 4,

** 50,

*** 2,

**** 6,

***** 19, and

****** 39

No significant differences were observed in maternal age <19 years (6.6% vs. 1.3%) or skin color (nonwhite) (45.5% vs. 40.8%) between the women admitted to public and private hospitals. Regarding previous clinical and obstetric conditions, comorbidities (chronic hypertension, gestational diabetes, and asthma) were not significantly difference between the two groups. The clinical manifestations of COVID-19-related symptoms were similar in both groups; however, the public hospital group had significantly higher rates of fever and tachypnea (Table 2).

**Table 2:** Clinical and laboratory manifestations at the inclusion of symptomatic women with confirmed COVID-19, according to the type of hospital care

Clinical and laboratory manifestations		Public hospitals n(%)	Private hospitals n(%)	p-value
Time since symptom onset (days)*				0.018
	<7 days	153(76.1)	43(60.6)	
≥7 days		48(23.9)	28(39.4)	
Fever >38 °C		95(44.6)	23(30.3)	0.041
Cough		137(64.3)	45(59.2)	0.513
Nasal congestion		37(17.4)	28(36.8)	0.001
Coryza		74(34.7)	35(46.1)	0.108
Dyspnea		86(40.4)	23(30.3)	0.155
Saturation of O_2_ < 95%**		20(9.6)	2(3.3)	0.180
Chest pain		13(6.1)	5(6.6)	1.000
Tachypnea***		64(32.2)	7(11.5)	0.003
Platelet count < 100,000/μl*****		12(10.2)	0(0.0)	0.359
Creatinine ≥ 1.2 mg/dL*****		15(13.0)	2(14.3)	1.000

ICU, intensive care unit; Missing information for

* 17,

** 20,

*** 29,

**** 156, and

***** 160

No significant differences between the two groups were noted in prematurity, occurrence of preeclampsia, mode of birth, birth weight, Apgar score, or ICU admission.

Considering maternal outcomes, table 3 shows that the public hospital group had a greater risk of SARS than the private hospital group (19.7% vs. 6.7%; *p* = 0.014). The public hospital group had a greater frequency of ICU admission than the private hospital group (16.5% vs. 8.0%). The same trend was observed for neonatal respiratory distress and neonatal death; however, these differences were not significant, possibly due to the small sample size. In total, 13 maternal deaths were recorded in the public hospital group, while no maternal deaths were noted among pregnant women treated at private hospitals.

**Table 3:** Pregnancy outcomes of symptomatic women with confirmed COVID-19, according to the type of hospital care

Pregnancy outcomes		Public hospitals n(%)	Private hospitals n(%)	p-value
Severe maternal outcomes*				
Maternal death		13(6.4)	0(0.0)	0.024
	Any SARS***	42(19.7)	5(6.7)	0.014
	Fetal death¹	4(2.6)	0(0.0)	0.577
	Preterm birth²	49(32.5)	10(22.7)	0.294
	Low birthweight³	36(27.7)	6(15.8)	0.201
	Apgar 5 min <7^4^	9(6.1)	0(0.0)	0.213
	Neonatal respiratory distress^5^	39(28.1)	4(9.3)	0.020
Admission to neonatal ICU^6^		44(30.8)	7(15.9)	0.082
	Neonatal morbidity^7^	19(16.2)	3(7.1)	0.229
Neonatal death^8^		5(3.5)	2(4.8)	0.658
APO WHO****^9^		61(39,9)	12(27,3)	0,178

* SARS, admission to NICU or maternal death

** intensive care unit

*** Severe acute respiratory syndrome

**** Adverse Perinatal Outcomes according to WHO definition Missing information for: ¹1, ²3, ³30, ^4^8, ^5^16, ^6^11, ^7^39, ^8^13, ^9^1

## Discussion

Our findings reveal significant differences in the sociodemographic profiles and maternal-fetal outcomes amongst women from the public and private healthcare systems. Women treated at public hospitals were more likely to have lower educational attainment, to report higher unplanned pregnancies, and not to be in a partnered relationship. They presented similar characteristics in age, skin color, or comorbidities.

Women assisted in public hospitals have higher rates of unplanned pregnancies. Similar data from a secondary analysis of a national multicenter cross-sectional study indicated that, in Brazil, only 32,5% were planned for that moment.^(9)^ Currently, the Unified Health System (SUS) offers several contraceptive methods. Among these methods, copper intrauterine devices are the most widely used worldwide (approximately 15%). However, its utilization in Brazil remains limited, with a reported rate of approximately 1.9%, according to Perioperative Nursing Data.^(9,10)^

Our findings revealed that among the groups, 13 maternal deaths were recorded. SARS and neonatal respiratory distress were worse in public hospitals, and no maternal deaths were recorded among the women in the private hospital group who were followed up during the study. These findings suggest disparities in our population and illustrate how these marked differences may be influenced by access to healthcare services and the quality of care provided. In Brazil, during the first wave of the pandemic, maternal mortality reached alarming levels, which is reflected in the maternal outcomes observed during our study.^(11)^ Recent evidence suggests that pregnant women of Black and minority ethnicities with COVID-19 are at a higher risk of mortality during pregnancy and the postpartum period. The same study indicates that 60% of the 10 women who died from direct COVID-19 were Black patients and ethnic minorities in the UK.^(12)^

Several publications have attempted to elucidate how existing structures of socioeconomic inequality, as well as pandemic-related social and economic stressors, influence SARS-CoV-2 infection during pregnancy and adverse birth outcomes.^(13,14)^ A systematic review by Mackey et al.^(15)^ revealed that African American/Black and Hispanic populations experience disproportionately higher rates of SARS-CoV-2 infection, hospitalization, and COVID-19-related mortality than non-Hispanic white populations do. In Brazil, socioeconomic inequalities, rather than age, health status, and other risk factors for COVID-19, have affected the course of the pandemic, with disproportionately adverse states and municipalities with greater socioeconomic vulnerability.^(16)^

Our first question was whether the maternal and perinatal outcomes of pregnant women with SARS-CoV-2 infection differ. In our study, approximately half of the symptomatic pregnant women were not tested for SARS-CoV-2 infection in the public network, whereas tests were conducted for more than 2/3 of the patients in the private network who had COVID-19 symptoms.

As suggested in our study, other countries may have presented similar numbers; as reported in a 2022 Lancet study, 64.8% (219/338) of pregnant and postpartum women who died from COVID-19 were admitted to critical care units, and 50.2% (160/319) received cardiopulmonary resuscitation. Maternal mortality levels might change through two hypothetical pathways: either through the aggravation of SARS-CoV-2 infection during pregnancy or through interruptions in access to maternity services.^(17)^ The lack of critical care might reflect barriers faced by pregnant women in accessing ICUs, such as the limited number of beds or the administrative referral process. The low admission rates may also be related to these countries’ overwhelmed healthcare systems and lack of critical care beds.

According to the Pan American Health Organization, a lack of access to timely care and interruptions in prenatal services caused an increase in maternal mortality in the Americas during the pandemic, with one in three pregnant women being unable to access care promptly. Hence, the challenges and inequalities we faced before the COVID-19 pandemic intensified and should be addressed.^(18)^

Our study was conducted in a pre-vaccination era. Intensive vaccine research and development have been undertaken to control the pandemic and prevent further outbreaks of the disease, which has overwhelmed healthcare systems worldwide. A systematic review of the safety, immunogenicity, and effectiveness of COVID-19 vaccines in pregnant women revealed that vaccination is capable of reducing maternal SARS-CoV-2 infection, maternal severe and critical disease, and perinatal death.

Safety data indicated that pregnant and lactating populations experienced vaccine-related reactions at rates similar to those of the general population.^(19,20)^

As this study comprises a secondary analysis of the multicenter REBRACO cohort, data were prospectively and uniformly collected across 15 Brazilian maternity hospitals. The comparison groups exhibited similar sociodemographic and clinical profiles; notably, there were no statistically significant differences regarding maternal age, skin color, or the presence of comorbidities. Inclusion criteria were stringent, incorporating only confirmed cases of COVID-19 with complete pregnancy outcomes. Additional strengths of the study include a low rate of loss to follow-up, reducing the risk of selection bias, and sufficient statistical power to support the analyses. Some limitations of our study include that our database did not allow us to determine if the outcomes were solely correlated with the type of care or if other associated variables were also involved. Another limitation was that some Brazilian regions were not included in the study. Additionally, this study had a limited sample size.

Changes in healthcare systems may persist long after the COVID-19 pandemic; these effects can delay diagnoses and impact maternal–fetal outcomes. Women who experience domestic violence, belong to minority groups, or live in less favorable conditions, with limited access to the internet and remote care, may be most affected by these changes.

## Conclusion

The COVID-19 pandemic has had direct and indirect effects on the health of pregnant women and perinatal outcomes. Our study highlights the need to strengthen access to quality care for vulnerable populations and prioritize pregnant women in public health strategies, including vaccination campaigns. Inequalities in the Brazilian healthcare system are profound and have serious consequences for maternal and neonatal outcomes. The COVID-19 pandemic has underscored these inequalities, emphasizing the importance of public policies aimed at reducing preventable mortality and achieving greater equity in health outcomes.

## Data availability

: The authors did not make the data from this article available in repositories prior to submission.
